# Gene Regulatory Network Inferences Using a Maximum-Relevance and Maximum-Significance Strategy

**DOI:** 10.1371/journal.pone.0166115

**Published:** 2016-11-09

**Authors:** Wei Liu, Wen Zhu, Bo Liao, Xiangtao Chen

**Affiliations:** College of Information Science and Engineering, Hunan University, Changsha, Hunan, 410082, China; King’s College London, UNITED KINGDOM

## Abstract

Recovering gene regulatory networks from expression data is a challenging problem in systems biology that provides valuable information on the regulatory mechanisms of cells. A number of algorithms based on computational models are currently used to recover network topology. However, most of these algorithms have limitations. For example, many models tend to be complicated because of the “large p, small n” problem. In this paper, we propose a novel regulatory network inference method called the maximum-relevance and maximum-significance network (MRMSn) method, which converts the problem of recovering networks into a problem of how to select the regulator genes for each gene. To solve the latter problem, we present an algorithm that is based on information theory and selects the regulator genes for a specific gene by maximizing the relevance and significance. A first-order incremental search algorithm is used to search for regulator genes. Eventually, a strict constraint is adopted to adjust all of the regulatory relationships according to the obtained regulator genes and thus obtain the complete network structure. We performed our method on five different datasets and compared our method to five state-of-the-art methods for network inference based on information theory. The results confirm the effectiveness of our method.

## Introduction

The rapid development of high-throughput technologies has produced extensive gene expression data, and mining useful cell function information from these data has become a crucial goal in systems biology [1,2]. Specific physiological activity in cells occurs at the gene expression level. This physiological activity results from the interaction of a large number of genes and biological molecules and is not controlled by the gene itself. The sophisticated regulatory relationships between genes are often depicted in the form of gene regulatory networks. Therefore, gene network inferences are crucial to identifying regulatory relationships and understanding regulatory mechanisms [3,4].

A gene network can be represented by a graph, G = {V, E}, where V and E represent the gene sets and the regulatory relationships between genes, respectively [5]. The graph depicts the gene network topology, which makes the interactions between genes more explicit. The main task of gene network inference methods is to recover accurate gene topologies from gene expression data and ensure their consistency with real gene networks. However, network inference is a challenging problem because of limitations in gene expression data; for example, when the number of samples is far less than the number of genes, an ill-posed network structure may result [6,7]. In addition, high noise and non-linear characteristics lead to inaccurate network inferences. Although these limitations may cause difficulties when performing network inferences, many inference methods of gene regulatory networks based on computational models have been reported in recent decades [8–12].

The Boolean network model is a simple model based on natural mechanisms. The expression value of each gene is either 0 or 1, and the interaction relationships between the genes are expressed by abstract Boolean logic, such as AND, OR, and NOT [13,14]. The Boolean network model was first proposed by Kauffman [15], who characterized the framework of the model. To reduce uncertainty in the data and model selection, Shmulevich et al. [16] extended the model and proposed the probabilistic Boolean network. Recently, a variety of computational methods have been introduced to improve Boolean network models, such as information theoretic [17,18], genetic algorithm [19,20], and literature-based methods [9]. Although Boolean networks are easy to implement, capturing complex system behaviors and recovering large-scale gene networks may be difficult using these network models [2,18].

The Bayesian network is a probabilistic graphical model that consists of a directed acyclic graph (DAG) and a conditional probability table (CPT). A DAG describes the causal relationships among a set of genes, and a CPT represents the conditional probability distribution of each gene based on its parent set [21]. The Bayesian network can be static or dynamic according to whether temporal expression profiles are used. Both static and dynamic Bayesian network modeling involve two components: structure learning and parameter learning. In this study, we mainly focused on the first component. Structure learning has become a challenging problem in recent decades. Two categories of methods can be used in structure learning: a constraint-based method [22,23], which adopts condition independence tests to capture the dependent relationships among genes; and a score-based method, which represents the measured probability of each structure from the given data. Recently, several score functions have been used, such as Akaike’s information criteria [24], Bayesian information criteria [25], and minimal description length [26,27]. Although Bayesian network modeling has certain advantages with regard to noise data and incomplete data, it also has drawbacks, with the major ones being the exponential growth of the search space size of a DAG with respect to the number of genes and the difficulty of recovering large-scale networks [28].

Network models based on information theory are widely applied in network inference and measure regulatory relationships between genes by the dependency of all gene pairs. Mutual information (MI) is used as the measure of dependency because of its excellent performance in capturing complex dependency. Many gene network inference algorithms based on MI have been proposed [29–36]. When recovering networks based on these methods, the mutual information matrix (MIM) is first calculated, and then different strategies are applied to distinguish between real edges and false edges. One of the earliest of these methods is the relevance network approach [29], in which a proportion of inaccurate edges are eliminated according to a given threshold. However, the method cannot be used to assess indirect interactions between genes. The CLR algorithm presents an MI score based on an empirical distribution [31]. The ARACNE algorithm is used to measure data processing inequality [32] to eliminate indirect interactions. Luo et al. [33] produced a new statistical learning strategy, MI3, for the detection of more complex three-way relationships. Zhang et al. [34] presented a network inference algorithm based on conditional mutual information (CMI) to distinguish non-linear dependence between genes. Meyer et al. [35] employed a new dependency paradigm by introducing a feature selection technique based on maximum relevance/minimum redundancy criteria. Villaverde et al. [36] presented the network inference algorithm MIDER, which is based on entropy reduction. Methods based on information theory can effectively capture non-linear dependency and apply it to large-scale networks. Nevertheless, many models only consider pairwise interactions between genes and ignore other characteristics of the network.

Inspired by the network model based on information theory and a feature selection method known as maximum relevance-maximum significance (MRMS) [37], we propose a novel information—theoretic network inference method based on the MRMS, in which the problem of network recovery is converted into a process whereby the regulator genes for each target gene are selected. Our proposed strategy differs from that of the MRMS in terms of feature selection because it fully considers network topology characteristics, such as relevance, modularity, and sparseness. Therefore, our strategy can effectively select the regulator genes of a target gene, and it can then be applied for network recovery. Our method is compared with typical methods based on information theory, and the results demonstrate that our method outperforms these models.

## Methods

Gene network inference can be implemented by selecting the regulator genes for each gene; however, the key problem with this method is that it fails to present an effective selection strategy. In this section, we propose a new MRMS strategy based on information theory and then present a compact network inference method called the maximum-relevance and maximum-significance network (MRMSn) method, which is based on MRMS. To clearly describe this method, we first introduce the motivations of the method, its relevance and significance in networks and the concepts of information theory, which are the foundation of our proposed method.

### Motivation

Generally, traditional gene network inference methods recover network topologies by using a computational model that ensures that the final network topology is the closest match to the gene expression data. However, building an appropriate model is difficult because of the “large p, small n” problem [38]. The fundamental task of network inference is to identify all of the underlying regulatory relationships between genes. Therefore, if each gene is sequentially treated as the target gene, then the problem of inferring a network involving n genes can be decomposed into n sub-problems, and the task involves selecting the regulator genes for the given target gene. Designing an effective selection strategy is the key to the sub-problem. At present, many selection strategies are being applied with feature selection techniques to resolve the classification problem. To a certain extent, the sub-problem of selecting the regulator genes for a given target gene can be considered a two-class classification problem. Hence, the feature selection technique is used to resolve the sub-problem in this paper.

To select the correct regulator genes for the target gene in each sub-problem, a selection criterion with a high discriminating power should be designed. Although a large number of different criteria have been applied to feature selection problems and each criterion attempts to select the optimal feature sets that are suitable for all applications, obtaining the optimal features is not realistic. Therefore, the optimal feature selection criterion in the above sub-problem should fully reflect the characteristics of the network topology.

For real network topology, relevance and significance are the most basic characteristics. Regulator genes should have a high relevance with the target gene and directly connect with the target gene. Thus, relevance can be used to search for the regulator genes of the target gene. However, certain genes with high numerical relevance with the target gene may not be directly connected with the target gene. Obviously, these genes, which are called redundant genes, are not actual regulatory genes. This situation may lead to difficulties in selecting regulator genes based on the relevance of the gene network and subsequently degrade the accuracy of the network inference. Hence, redundant genes must be removed. Significance is another variable for determining whether a gene is the true regulator gene of the target gene. Regulator genes should have high significance with the module, which is composed of the target gene and the regulator genes. Therefore, a strategy that combines relevance and significance may effectively remove redundant genes and thereby improve the accuracy of the network inference.

### Relevance and significance

Relevance and significance are the most basic characteristics of a network. Relevance is usually used to measure the dependency between genes in the network, and significance reflects the influence of the genes on the network. For a network with a target gene and corresponding regulator genes, the relevance and significance can be defined as follows.

Let *G* = {*g*_1_,…,*g*_*n*_} denotes the set of *n* genes of a given microarray dataset, which is used to infer the network. The relevance of gene *g*_*i*_ with respect to the target gene *g*_*c*_ is defined as follows:

$$\gamma_{g_{i}}=R\left(g_{i},g_{c}\right)$$ (1)

where *R*(·) is a measurement that represents the dependency between two genes. Mutual information is one of the typical measures to define dependency of variables and we use it to characterize the relevance of genes in this paper.

Let *U* = {*g*_1_,…,*g*_*r*_},*U* ⊂ *G* be the set of *r* selected genes. For the target gene *g*_*c*_(*g*_*c*_ ∈ *G*\*U*), the significance of gene *g*_*i*_ among *U* has the following form:

$$\varphi_{g_{i}}=S_{U}\left(g_{i},g_{c}\right)=\left|E_{U}\left(g_{c}\right)-E_{U\backslash \{g_{i}\}}\left(g_{c}\right)\right|$$ (2)

where *E*(·) represents an energy estimation measure. The description of the energy measure can be different in different contexts. Entropy is an effective measurement to characterize energy dispersal, and it can be used to measure information content of variable in information theory. Thus, we use entropy to measure the energy estimation of genes in this study. Basically, the significance defined in Eq (2) reflects the change in energy estimation when a gene *g*_*i*_ is removed from the gene set *U*.

### Information theory

Information theory was proposed by Claude E. Shannon and has become widely used in applied mathematics, electrical engineering and computer science. Entropy and MI are two fundamental concepts in information theory that are vital for the relationships and interpretation of data.

Entropy is a measure of the average uncertainty of a random variable. Here, *X* represents a discrete random variable with alphabet *χ* and *p*(*x*) represents the probability distribution function of *X*. The entropy of a random variable *X* is defined as follows:

$$H\left(X\right)=-\sum_{x\in X}p\left(x\right)\text{log }p\left(x\right)$$ (3)

where the log is to the base 2 and the entropy is expressed in bits.

Because of the discrete random variable *Y* and the conditional distribution function *p*(*y*|*x*), the conditional entropy *H*(*Y*|*X*) is defined as follows:

$$H\left(Y|X\right)=-\sum_{x\in X,y\in Y}p\left(x,y\right)\text{log }p\left(y|x\right)$$ (4)

The definition of conditional entropy can be extended to multiple random *X*_1_,…,*X*_*i*_ and is defined as follows:

$$H\left(Y|X_{1},\ldots ,X_{i}\right)=-\sum_{y\in Y,x_{1},\ldots ,x_{i}\in X}p\left(y,x_{1},\ldots ,x_{i}\right)\text{log }p\left(y{|x}_{1},\ldots x_{i}\right)$$ (5)

*H*(*Y*|*X*_1_,…,*X*_*i*_) is monotonically decreasing, and it follows that

$$H\left(Y|X_{1},\ldots ,X_{i}\right)\leq H\left(Y|X_{1},\ldots ,X_{i-1}\right)$$ (6)

MI is used to describe information that a random variable shares with another random variable and represents a measure of the dependency relationship between the two variables. The MI of two random variables *X* and *Y* is defined as follows:

$$I\left(X,Y\right)=H\left(Y\right)-H\left(Y|X\right)=\sum_{x\in X}\sum_{y\in Y}p\left(x,y\right)\text{log}\left(\frac{p\left(x,y\right)}{p\left(x\right)p\left(y\right)}\right)$$ (7)

where *p*(*x*,*y*) is the joint probability distribution function of *X* and *Y* and *p*(*x*) and *p*(*y*) are the marginal probability functions of *X* and *Y*, respectively.

### MRMS strategy

In real network topologies, each regulator gene should have high relevance to the target gene. Hence, the identification of the regulator genes for a target gene is to select a gene set *S* with *K* regulator genes {*g*_*i*_}that have the greatest relevance to the target gene *g*_*c*_. Let *G*_*c*_ = {*g*_1_,…,*g*_*c*−1_,*g*_*c*+1_,…,*g*_*n*_} represents the candidate gene set containing all genes in *G* except for target gene *g*_*c*_. MI is used to represent the relevance between the target gene and the regulator gene. The regulator genes of target gene *g*_*c*_ are the genes that satisfy Eq (8), which is called the maximum relevance criterion.

$$\text{max}R\left(S,g_{c}\right),\;R=I\left(\left\{g_{i},i=1,\ldots ,K\right\},g_{c}\right)$$ (8)

Obviously, when *K* equals 1, the selected gene is the gene that maximizes *I*(*g*_*i*_,*g*_*c*_)(*g*_*i*_∈ *G*_*c*_). When *K*>1, a simple selection scheme is adopted: suppose *V* is a gene set with m-1 selected regulator genes, the *m*th regulator gene *g*_*m*_ is selected by maximizing *R*(·) in Eq (9).

$$R\left(g_{m},g_{c}\right)=\left\{I\left(g_{m},g_{c}\right);g_{m}\in G_{c}\backslash V\right\}$$ (9)

Although the regulatory genes should have high relevance to the target gene, not all genes with high relevance to the target gene are true regulator genes. A likely occurrence is that certain genes selected on the basis of the maximum relevance criterion will not be directly connected with the target gene, and the genes will connect with target gene through a true regulator gene. Obviously, these genes, which are called redundant regulator genes, are not the correct regulator genes. Redundant regulator genes can degrade the accuracy of the network inferences and must therefore be removed.

Modularity is an important characteristic of regulatory network topologies. A module is composed of clustered genes. Compared with other genes outside of the module, the genes inside the module have more associations with each other. Typically, a module is a relatively balanced system in a real network, and the real gene members in this system have different influences on the system according to their differing importance levels. Theoretically, the members outside of the system will have no influence on the system. This influence is called the significance of the gene in the module. A module composed of a target gene and corresponding regulator genes can be a special module in which the target gene is the core of the module, which is regulated by all of the regulator genes. Every regulator gene provides a different contribution to the information of the target gene. Obviously, the significance of the gene can be used to discriminate if the gene is the true regulator gene for the target gene. Thus, defining significance in the special module is key for understanding the modularity of regulator genes and removing redundant regulator genes. Entropy is an effective method of measuring the variable amount of information in information theory and can be used to measure energy dispersal. Based on Eqs (2) and (6), we considered using an entropy reduction method to measure the significance.

For a target gene *g*_*c*_ and the gene set *V* with m-1 genes, the significance of gene *g*_*m*_ has the following form:

$$S(g_{m},g_{c})=S_{VU\{g_{m}\}}\left(g_{m},g_{c}\right)=H\left(g_{c}|V\right)-H\left(g_{c}|VU\{g_{m}\}\right)$$ (10)

Clearly, for target gene *g*_*c*_, the regulator gene should be the top-ranked gene in the significance score (see Eq (10)). This strategy is called the maximum significance method, which is described as follows:

$$\underset{g_{m}\in G_{c}\backslash V}{\text{max}}S\left(g_{m},g_{c}\right)$$ (11)

Based on the above description of two criteria, we note that the maximum relevance criterion used to select for regulator genes may introduce redundant regulator genes. To remove the redundant regulator genes, we consider the maximal significance strategy because the correct regulator genes tend to score higher on significance. The strategy that combines the maximal significance strategy with the maximum relevance criterion is called the MRMS. Ideally, the correct regulator genes should have high relevance to the target gene and high significance in the module. Thus, the following form is provided to optimize *R* and *S* simultaneously:

maxΦ(R,S),Φ=R+S (12)

For feature selection, combining two strategies to select the optimal regulator genes ensures that the selected regulator genes have maximal relevance and significance to the target gene. Therefore, identifying the regulator genes of a target gene is the same as selecting *K* regulator genes from *G*_*c*_ by maximizing Φ(·) in Eq (12). In practice, this goal cannot be achieved, and only near-optimal regulator genes can be obtained. Inspired by the maximal relevance-minimal-redundancy criterion and considering the degree of relative importance between relevance and significance, another form is derived from Eq (12). Suppose a gene set *V* with m-1 regulator genes has been selected. When selecting the *m*th gene, the selected gene *g*_*m*_ has the following form:

$$\underset{g_{m}\in G_{c}\backslash V}{\text{max}}\left[\alpha I\left(g_{m},g_{c}\right)+\left(1-\alpha \right)\left(H\left(g_{c}|V\right)-H\left(g_{c}|VU\{g_{m}\}\right)\right)\right]$$ (13)

Based on Eq (13), we adopt the following first-order incremental search algorithm to select the regulator genes for the target gene *g*_*c*_.

Step 1: The candidate gene set *G*_*c*_ and the set of the selected regulator genes *V* are initialized, and *G*_*c*_ = *G* − *g*_*c*_ and *V* = *ϕ* are set.
Step 2: The relevance values *I*(*g*_*i*_,*g*_*c*_) between the target gene *g*_*c*_ and each candidate gene *g*_*i*_∈ *G*_*c*_ are calculated, and the relevance values are ranked in descending order.
Step 3: The candidate gene with the largest relevance value is selected as the first gene of *V* and removed from *G*_*c*_.
Step 4: In the remaining genes of *G*_*c*_, a gene *g*_*m*_ that maximizes Eq (13) is selected. If the score in Eq (13) of the selected gene lies above the given score threshold *T*_0_, then the gene is inserted into *V* and removed from *G*_*c*_. Otherwise, the selection procedure is terminated.
Step 5: Step 4 is performed again until the number of selected genes in *V* is more than the given number *K*; otherwise, the selection procedure is terminated.

Note that the parameter *α* represents the weight of the relevance, and it is used to adjust the importance between the relevance and significance. Parameter *K* represents the number of selected regulator genes. Considering the sparseness of the network, the number *K* is set to ⌈log_2_ *n*⌉, where *n* is the number of genes in the network.

### Gene network inference based on the MRMS strategy

Inferring a gene network involves identifying all of the regulatory relationships between genes. In the above section, we describe an MRMS strategy that helps to identify the regulatory relationships of a target gene. The remaining work involves inferring the gene network topology based on this strategy. We propose a novel regulatory network inference method called MRMSn, which recovers gene networks through the following three stages.

The first stage is the regulatory relationship initialization. In this stage, a MIM is built according to Eq (7), and this MIM can reflect the likelihood of most direct regulatory relationships among genes. Generally, a greater value corresponds to a higher likelihood of a direct regulation relationship. However, the sparsity of the network means that only a few regulatory relationships occur between genes. Hence, a regulatory relationship with small values in the matrix must be removed. The most commonly used removal method is the selection of a unified threshold to remove false regulatory relationships. If the MI value is less than the threshold value, then the MI value is set to zero and the corresponding regulation relationship is removed. However, the numerical range of the MI value for each gene is different. When the unified threshold is too large, all regulatory relationships of certain genes may be lost, which is an undesired result. Therefore, in the network inference algorithm, we provide different thresholds for the regulatory relationships of different genes, and the threshold for the regulatory relationship of a given gene is set according to the MI values between the given gene and other genes. For a MIM *M*(*n*×*n*), *M*_*i*_ denotes the MI values between gene *g*_*i*_ and other genes. We choose parameter *θ* as the threshold for initializing regulatory relationships because it satisfies *θ* = *ε*×max(*M*_*i*_), where *ε*∈(0,1). Parameter *ε* is typically set to a small value; specifically, it is set as 0.01 by default.

The second stage involves selecting the regulator genes for all genes. Each gene in a given gene dataset will in turn be a target gene, and the regulator genes of each target gene are selected using the MRMS strategy.

The last stage is to adjust all of the regulatory relationships and build a complete network structure. Although all of the regulatory genes of each target gene have been obtained, we cannot accurately obtain the complete network. A case that should be considered is when gene *g*_*i*_ is the regulator gene of gene *g*_*j*_ and gene *g*_*j*_ is not the regulator gene of gene *g*_*i*_. To resolve this problem, we provide a constraint to adjust the regulation relationships to obtain the complete network structure. The constraint is defined such that the regulatory relationship between gene *g*_*i*_ and gene *g*_*j*_ only occurs if gene *g*_*i*_ is the regulator gene of gene *g*_*j*_ and gene *g*_*j*_ is the regulator gene of gene *g*_*i*_. After the regulation relationships are adjusted according to this constraint, certain target genes may not have a regulator gene. To ensure that each target gene has at least one regulator gene, the first selected regulator gene of the target gene in the MRMS strategy is regarded as the regulated gene of the target gene.

The gene network inference method MRMSn is summarized in Table 1.

**Table 1:** MRMSn for network inference.

Input: Microarray data *G* = {*g*_1_,…,*g*_*n*_}
The number of the selected regulator gene *K*
The weight of network relevance *α*
The threshold of initializing regulatory relationships *ε*
The threshold of scoring in MRMS *T*_0_
Output: A gene network
1: Construct a MI matrix *M* according to Eq (7).
2: Adjust MI matrix *M* using the threshold *ε*
3: for each gene *g*_*c*_, *c*←1 to n do
4: Select K regulator genes of gene *g*_*c*_ using MRMS criterion
5: end
6: Adjust the regulation relationship using the constraint.
7: Return the gene network.

### Parameter tuning problem

The parameter *α* plays an important role in gene regulatory network inferences based on the MRMS strategy because it adjusts the degree of relative importance between the relevance and significance in the model. If *α* increases, the importance of the relevance increases, but the importance of the significance decreases. Thus, this parameter directly influences the performance of the proposed method.

To determine the optimum value of *α*, an optimization method based on the local density is provided. The local density *ρ*_*c*_ of the target gene *g*_*c*_ is defined as follows:

$$\rho_{c}=\sum_{j=1}^{n}\chi \left(I\left(g_{c},g_{j}\right)-d\right)$$ (14)

where *χ*(*x*) = 1 if *x* ≥ 0 and *χ*(*x*) = 0 otherwise and *d* is a cutoff distance. Basically, *ρ*_*c*_ is equal to the number of genes with which the relevance of gene *g*_*j*_ is more than *d*, which reflects the approximate number of regulator genes of gene *g*_*c*_. Clearly, the value of *ρ*_*c*_ will be affected by *d*, and the value of *d* should be determined according to the datasets. Let $sm_{g_{c}}$ (*g*_*c*_ ∈ *G*) denote the sum of the MI between gene *g*_*c*_ and any other genes. For each target gene *g*_*c*_ ∈ *G*, the $sm_{g_{c}}$ is calculated and gene *g*_*γ*_ that satisfies Eq (15) is selected.

$$g_{\gamma }=\underset{g_{c}\in G}{\text{arg min}}\left\{sm_{g_{c}}\right\}$$ (15)

Intuitively, the number of regulator genes of gene *g*_*γ*_ may be lowest among all of the genes. In a sparse network, this number is typically one, although in a complex method, it is more than one. Thus, the value of *d* can be calculated as follows:

$$d=\underset{g_{j}\in G}{\text{max}}I\left(g_{\gamma },g_{j}\right)$$ (16)

Basically, *d* in Eq (16) is equal to the highest MI value between gene *g*_*γ*_ and any other gene. When the value of *d* is used in Eq (14), the density of certain genes that present the highest MI value among other genes with values lower than *d* in Eq (16) may be zero. Thus, when the density of certain genes is zero, the value of *d* is adjusted as follows:

$$d=\frac{1}{n-1}\sum_{j=1}^{n}I\left(g_{\gamma },g_{j}\right)$$ (17)

After the cutoff distance *d* is calculated, the density *ρ*_*c*_ of each gene can be obtained. The parameter *α* is defined as follows:

$$\alpha =\frac{\sum_{i=1}^{n}\Gamma \left(\left\lceil {\text{log}}_{2}\;n\right\rceil -\rho_{i}\right)}{n}$$ (18)

where Γ(*η*) = 1 if *η*>0; otherwise, Γ(*η*) = 0. Considering that relevance and significance are the basic characteristics of the network, a constraint in which the value of *α* is varied from 0.1 to 0.9 is included. If *α* is less than 0.1, the final *α* is set to 0.1, and if *α* is more than 0.9, the final *α* is set at 0.9.

The above optimization method is applied to each dataset. The optimum value of *α* is 0.25 for Reaction chain with 4 species data, 0.90 and 0.10 for the DREAM3 10 genes data and DREAM3 50 genes data, respectively, and 0.20 and 0.78 for the IRMA benchmark data and SOS data, respectively.

Parameter *T*_0_ represents the threshold of the score in Eq (13) and is used to select regulator genes. A gene is considered to be the regulator gene of a target gene when the gene score exceeds *T*_0_. During the selection process, all scores in Eq (13) can be observed, and they are obtained in the process of selecting the regulator gene for each target gene. Based on extensive experiments, the value of *T*_0_ is set to 70% of the maximum score. Thus, the optimum values of *T*_0_ are 0.55, 0.09, 0.12, 0.51 and 0.18 for Reaction chain with 4 species data, DREAM3 10 gene data, DREAM3 50 gene data, IRMA benchmark data and SOS data, respectively.

### Computational complexity

In this section, we detail the computational complexity of the proposed method. Let *n* be the number of genes in the network and *n*′ be the number of selected regulator genes in already-selected gene set. All of the relevance values of the target genes are obtained by calculating the mutual information matrix, which requires a *o*(*n*^2^) time complexity. The gene with the largest relevance value to the target gene is selected in step 3 of the MRMS strategy, and this step requires *o*(*n*−1). After obtaining the first regulator gene, the genes in the candidate gene set are selected based on the maximum-relevance and maximum-significance strategy in step 4, and this step requires a *o*(*n*−*n*′) time complexity. Because the top *K* regulator genes are selected for the target gene, the selection procedure in step 4 is executed *K*−1 times. In the last stage of the MRMSn, a time complexity of *o*(2*Kn* + *n*) is required to adjust the relationships and build a complete network structure. Thus, when inferring the network with *n* genes, the overall computational complexity is *o*(*n*^2^)+*o*(*n*((*n*−1)+(*K*−1)(*n*−*n*′)))+*o*(2*K*+n). Because the number of total genes is significantly larger than the number of selected regulator genes (*n*>>*n*′), the computational complexity can be approximated as *o*(*Kn*^2^).

## Experiments

To evaluate the performance of the proposed MRMSn, we compare the method with five algorithms: CLR, ARACNE, MRNET, MI3 and MIDER. The first two methods are based on information theory and are widely used in network inference. MRNET and MI3 are regarded as two typical methods in which the regulation relationships involving other regulator genes are identified. MIDER can detect interactions with entropy reduction. CLR, ARACNE and MRNET can be implemented in the R package MINET. To avoid unfair comparisons, we chose the default parameter eps = 0.0 in ARACNE [39]. For CLR and MRNET, we calculate the true positive rate (TPR) and the false positive rate (FPR) at different thresholds and select the optimum threshold, for which (TPR-FPR+1) is the maximum in the method. For the datasets in Table 2, the thresholds of CLR are set to 0.4959, 0.0, 0.1233, 0.2505, 0.25 and the thresholds of MRNET are set to 0.0, 0.0114, 0.0105, 0.0577, and 0.0. MI3 is also implemented in R with the package mi3. MIDER and the proposed MRMSn are performed in the MATLAB environment on a personal computer with an Intel core i7 (2.2 GHz) and 16 GB of RAM. The proposed MRMSn does not provide network directions, and the network direction in MI3 and MIDER is not considered.

**Table 2:** The details of the data sets used in our experiments.

Datasets	Variables	Samples	Type	network nodes	network edges
Reaction chain with 4 species	4	10	Simulated	4	3
DREAM3 10 genes	10	10	Simulated	10	10
DREAM3 50 genes	50	50	Simulated	50	77
IRMA benchmark	5	125	Real	5	7
S0S	9	9	Real	9	24

### Data sets

Five datasets were used in the experiments to evaluate the performance of the method. All of the datasets included simulated data and real data obtained from previous studies [40–43]. Details of the datasets are given in Table 2.

Reaction chain with 4 species data [40] is a small reaction pathway that contains 10 samples for 4 variables. The true network is composed of 4 nodes and 3 edges.

DREAM3 10 gene data [41] represent a yeast network that contains 10 samples for 10 genes. The true network is composed of 10 nodes and 10 edges.

DREAM3 50 gene data [41] represent a yeast network that contains 50 samples for 50 genes. The true network is composed of 50 nodes and 77 edges.

IRMA benchmark data [42] represent a yeast synthetic network that contains 125 samples for 5 genes. The true network is composed of 10 nodes and 33 edges.

S0S data [43] represent an *Escherichia coli* network that contains 9 samples for 9 genes. The true network is composed of 9 nodes and 24 edges.

### Evaluation metrics

Our method must be compared with other methods that use different metrics to assess its performance. Therefore, we used the true positive rate (TPR), false positive rate (FPR), positive predictive value (PPV), and accuracy (ACC). We let true positives (TP), true negatives (TN), false positives (FP), and false negatives (FN) denote the numbers of true positives, true negatives, false positives, and false negatives, respectively. These measures are defined as follows:

$$TPR=\frac{TP}{TP+FN}$$ (19)

$$FPR=\frac{FP}{FP+TN}$$ (20)

$$PPV=\frac{TP}{TP+FP}$$ (21)

$$ACC=\frac{TP+TN}{TP+FP+TN+FN}$$ (22)

### Experimental results

To fairly evaluate the performance of our proposed method, we compared it with other methods using simulation data and real data. It was important to consider network size when choosing these datasets. The number of network nodes varies from 4 to 50 in our datasets. Network sparsity is an important aspect that should be considered. Network datasets that have different degrees of sparsity were chosen to reflect the scalability of our method. The experiment process for different datasets is described in detail in the following subsection.

#### Reaction chain with 4 species

**Fig 1:**
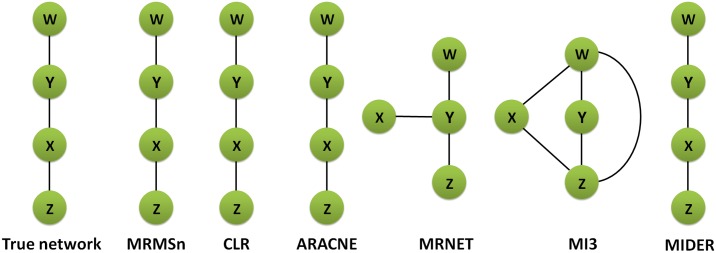
Comparison of different methods on reaction chain with 4 species dataset.

The proposed method was tested on a reaction chain that includes data for 4 species to verify how well it performs in special networks, such as linear chain networks. In this experiment, we chose 0.25 as the weight of the network relevance and 0.55 as the threshold of scoring in the MRMS according to the optimization methods proposed above. We set 2(⌈log_2_ 4⌉) as the number of the selected regulator genes. Fig 1 shows the network structure of different methods to facilitate a visual observation of the network topology. We found that MRMSn, CLR, ARACNE and MIDER can infer the same network topology with the true network, although redundant edges are produced in MRNET and MI3. To further reflect the efficiency of our method, the performance of MRMSn with regard to evaluation metrics was compared with the listed methods. Table 3 presents the comparison results and shows that CLR, ARACNE, MIDER and MRMSn can identify all of the correct edges and do not produce redundant edges (TP = 3, FP = 0). For MRNET and MI3, the values of ACC are 0.833 and 0.333, respectively, and these values are less than that of MRMSn (ACC = 1), which indicates that the proposed method performs better than the CLR and MRNET methods.

**Table 3:** Comparison of the performance by different methods on the reaction chain with 4 species dataset.

	TP	FP	TPR	FPR	PPV	ACC
CLR	3	0	1	0	1	1
ARACNE	3	0	1	0	1	1
MRNET	3	1	1	0.333	0.750	0.833
MI3	2	3	0.667	1	0.400	0.333
MIDER	3	0	1	0	1	1
MRMSn	**3**	**0**	**1**	**0**	**1**	**1**

#### DREAM3 challenge network

The DREAM project provides benchmarks and tools that can be used for the rigorous testing of gene network inference methods. To assess the ability of the MRMSn to analyze simulation data, we tested the proposed method with the DREAM3 challenge, which provides three sub-challenges with networks of sizes 10, 50, and 100. Only the results of the first two sub-challenges are presented here.

**Fig 2:**
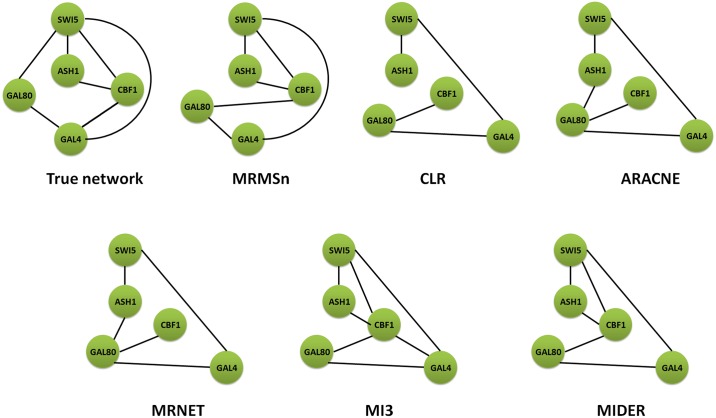
Comparison of different methods on DREAM3-10genes challenge.

First, we tested the MRMSn method with the yeast dataset that contains 10 samples for 10 genes. We set 0.90 as the weight of the network relevance and 0.09 as the threshold of scoring in MRMS. The number of the selected regulator genes for the target gene can be calculated as 4(⌈log_2_ 10⌉). The network topologies of the different methods for the dataset are shown in Fig 2, which shows that the MRMSn method can select all of the correct edges except for edge G4–G9. In addition, edge G2–G9 is the only redundant edge. The MRMSn method clearly performs better than the other methods. To support this finding, Table 4 presents a comparison of the results in the evaluation metric among the different methods. MRMSn can obtain 9 correct edges (TP = 9), whereas the other methods only obtain 6–8 correct edges. Note that MIDER cannot be used with the dataset. In addition, MRMSn only produces 1 redundant edge (FP = 1), whereas the other methods produce 6–12 redundant edges. The accuracy of our method is 0.956, which is much higher than the accuracy values of the other methods.

**Table 4:** Comparison of the performance by different methods on DREAM3-10 genes dataset.

	TP	FP	TPR	FPR	PPV	ACC
CLR	6	10	0.600	0.286	0.375	0.689
ARACNE	6	6	0.600	0.171	0.500	0.778
MRNET	6	12	0.600	0.343	0.333	0.644
MI3	8	6	0.800	0.171	0.571	0.822
MIDER	------	------	-------	------	-------	------
MRMSn	**9**	**1**	**0.900**	**0.029**	**0.900**	**0.956**

Then, MRMSn was tested on the yeast dataset that contains 50 samples for 50 genes. In the experiment, we chose 0.10 as the weight of the network relevance and 0.12 as the threshold of scoring of the MRMS. The number of the selected regulator genes for the target gene was 6(⌈log_2_ 50⌉). In the experiment, we observed that FP increased dramatically as TP increased in the other five methods. The experimental results are shown in Table 5 and indicate that the MRMSn method can identify 21 correct edges and that it includes only 17 redundant edges (TP = 21, FP = 17). In addition, this method can perform well with other metrics, especially PPV and ACC (PPV = 0.553, ACC = 0.940). Our approach clearly performs better than the other tested methods.

**Table 5:** Comparison of the performance by different methods on DREAM3-50 genes dataset.

	TP	FP	TPR	FPR	PPV	ACC
CLR	19	115	0.247	0.144	0.103	0.818
ARACNE	13	125	0.170	0.109	0.094	0.846
MRNET	21	215	0.273	0.187	0.089	0.779
MI3	21	68	0.273	0.059	0.236	0.899
MIDER	4	79	0.052	0.069	0.048	0.876
MRMSn	**21**	**17**	**0.273**	**0.015**	**0.553**	**0.940**

#### IRMA benchmark network

**Fig 3:**
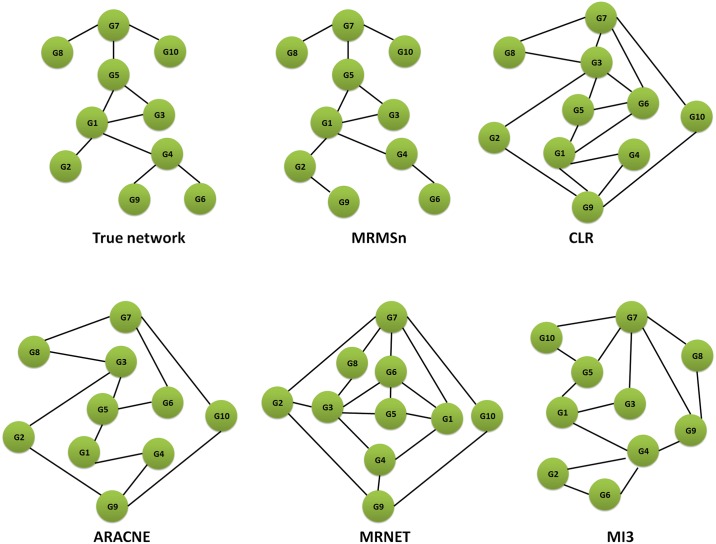
Comparison of different methods on IRMA benchmark.

To verify the effectiveness of the MRMSn method on real gene expression data, the method was tested on IRMA benchmark data and used to infer the IRMA benchmark network, which is a true yeast synthetic network. When the method was performed, the weighting of network relevance and the threshold of scoring in the MRMS strategy were set as 0.2 and 0.51, respectively. The number of regulator genes selected for the target gene was set to 3(⌈log_2_ 5⌉). Fig 3 presents the network structure of different methods using the IRMA benchmark dataset. The figure shows that the MRMSn correctly selects five of the seven true edges. However, SWI5–GAL80 and CBF1–GAL4 are lost and GAL80–CBF1 is a redundant edge. Similar comparison results with different methods can be obtained for the IRMA benchmark dataset. Table 6 shows that our method and MIDER perform better than CLR, ARACNE and MRNET, whereas MI3 (TPR = 0.857, FPR = 0.333) performs better than the MRMSn (TPR = 0.714, FPR = 0.333). Nevertheless, our MRMSn method performs well with the IRMA benchmark dataset (PPV = 0.833, ACC = 0.700).

**Table 6:** Comparison of the performance by different methods on IRMA benchmark dataset.

	TP	FP	TPR	FPR	PPV	ACC
CLR	3	1	0.427	0.333	0.750	0.500
ARACNE	3	2	0.429	0.667	0.600	0.400
MRNET	3	2	0.429	0.667	0.600	0.400
MI3	6	1	0.857	0.333	0.857	0.800
MIDER	5	1	0.714	0.333	0.833	0.700
MRMSn	5	1	0.714	0.333	0.833	0.700

#### SOS network in *E*. *coli*

The SOS network is a signal pathway in the DNA repair system. It is inferred from real gene expression data and is frequently used to test the effectiveness of network inference methods. Here, we test the MRMSn on the network of *E*. *coli*. We set the weighting parameter of network relevance as 0.78 and the threshold of scoring as 0.18. The number of regulator genes selected for the target gene was 4(⌈log_2_ 9⌉). Table 7 presents the results of the six methods applied to the SOS network in the *E*. *coli* dataset. The results show that the MRMSn performs better than all of the other methods except MRNET in terms of accuracy. Although our method did not identify the highest number of correct edges, it produced the fewest redundant edges of all of the methods. In addition, MIDER cannot be used with the SOS dataset. Although the performance of our method is poor for the TPR (TPR = 0.417), it performs better than the other methods for the FPR and PPV (FPR = 0.167, PPV = 0.833). Compared with previous experiments with other datasets, the effectiveness of the MRMSn on the SOS network is not ideal. The principal reason for this finding is related to the characteristics of the SOS network because most genes have 6–8 edges. However, the number of regulator genes selected for the target gene in the MRMSn is set to 4, which causes our method to overlook correct edges. Although the experimental results are not ideal, Table 7 shows that our method is nonetheless more effective than all of the other tested methods except MRNET.

**Table 7:** Comparison of the performance by different methods on SOS dataset.

	TP	FP	TPR	FPR	PPV	ACC
CLR	12	5	0.500	0.417	0.706	0.528
ARACNE	7	3	0.292	0.250	0.700	0.444
MRNET	17	6	0.708	0.500	0.739	0.639
MI3	9	5	0.375	0.417	0.643	0.444
MIDER	-------	------	------	------	------	------
MRMSn	10	**2**	0.417	**0.167**	**0.833**	0.556

### More performance evaluation

MRMSn was implemented to evaluate the performance based on the optimum threshold. To test the efficiency of the method on a variety of thresholds, we calculated the area under the receiver operating characteristic curve (AUROC) of the method on five datasets. We also compared the AUROC value of MRMSn with that of CLR, ARACNE, MRNET, MIDER and MI3. Except for MI3, these inference methods have some tunable parameters. Consequently, we did not show results for MI3. Table 8 presents the AUROC value results of the five methods for five datasets. From the table, we can see MRMSn, CLR, ARACNE and MIDER perform very well on Reaction chain with 4 species data, and the AUROC values reach 1. For other datasets, we can observe that our method performs better than all other methods. In particular, the AUROC value on DREAM3 10 gene data reaches 0.944, which is significantly more than other methods. All the results show the effectiveness and efficiency of the MRMSn method.

**Table 8:** AUROC scores for five datasets using different methods.

Datasets	CLR	ARACNE	MRNET	MIDER	MRMSn
Reaction chain with 4 species	1	1	0.889	1	1
DREAM3 10 genes	0.654	0.709	0.629	------	0.944
DREAM3 50 genes	0.542	0.531	0.530	0.509	0.690
IRMA benchmark	0.476	0.476	0.500	0.667	0.667
S0S	0.559	0.519	0.559	------	0.660

## Discussion

In this paper, we emphasize that the feature selection technique can be effectively applied to recover gene regulatory networks and obtain satisfactory accuracy. Our method converted the recovery of the gene regulatory network to the selection of the regulator genes of all of the target genes based on a feature selection strategy. The process of selecting the regulator genes is independent for each target gene. Therefore, this approach enables the use of parallel technologies to recover gene regulatory networks, and it is advantageous for inferring large-scale gene networks.

The MRMS selection criterion was proposed to measure the relevance between a target gene and regulator genes and simultaneously maintain the significance in local network structures. MIDER is also a network inference method based on the significance strategy, which refines the existing edges based on the significance strategy (entropy reduction) and infers the network structures. However, MRMSn refines the edges among genes according to a score function, which combine the relevance and significance strategy. Furthermore, there are some difference between MRMSn and MIDER: for each target genes, MRMSn stops adding genes when a predefined maximum K is met, while MIDER uses a different stopping criterion; and MSMRn uses local density to calculate the threshold for different datasets, while MIDER gives the threshold based on entropy reduction. All the experimental results show that the MRMSn can effectively infer gene regulatory networks in most cases. However, as the number of genes increases, calculating the criterion, which involves MI and multivariate conditional entropy, will lead to inaccuracies and difficulties when inferring large-scale networks.

Simulation data and real data were applied in our experiments. For the simulation data, the accuracy of the MRMSn is satisfactory and most of the correct regulation relationships can be identified. MRNET is another network inference method based on the feature selection technique. However, our method stresses the importance of significance in modularity in addition to relevance. The comparison indicates that the MRMSn is better than MRNET and the other methods for certain evaluation metrics. Significance can also eliminate redundant edges caused by the maximum relevance strategy. For the real data, the performance of the MRMSn outperformed most of the other methods, although not with perfect accuracy. These drawbacks may have been due to various causes, including the strong noise that may be contained in real data, which might lead to failures in selecting the real relationships between genes. Another cause involves the selection of parameter *K* in the MRMSn scheme. The number of regulator genes for a target gene in certain real networks may be more or less than the parameter provided in our paper, which will affect the accuracy of the regulator selection. For example, in the SOS network of *E*. *coli*, the maximum number of regulator genes for the target gene can reach 6, which is more than the given parameter. Thus, for the given parameter, certain regulation relationships will be lost, which was confirmed by the experimental results.

In the experiments, we found that the MRMSn works better in simple networks than in complex networks, especially for predicting certain special networks. For example, all of the regulatory relationships can be found in the reaction chain with 4 species. However, our results show that certain regulatory relationships can be lost in more complex networks, such as in the complicated SOS networks, in which almost all of the genes have connections with each other. This finding is still determined by parameter *K*. Although this type of network is uncommon and most networks still follow the sparsity of networks, the MRMSn is limited because it may not be able to estimate the optimal parameter *K*. Therefore, future work will focus on developing theoretical estimations for the number of regulators for target genes.

## Conclusions

In this paper, we developed the novel method MRMSn for inferring gene regulatory networks. The method treats the problem of network recovery as a problem of selecting regulator genes for each gene. We proposed an MRMS algorithm based on information theory. The definition of relevance and significance is the key to this algorithm, and these parameters are measured by MI and the reduced entropy of the target gene. A first-order incremental search algorithm can be used to search for regulator genes. After obtaining regulator genes for all of the target genes, a strict constraint is adopted to adjust the regulatory relationships and obtain the complete network structure. Five standard datasets were applied to test the methods, and we found that the MRMSn method can infer network structures efficiently on both simulated and real data. We compared our method with the CLR, ARACNE, MRNET, MI3 and MIDER methods and found that the performance of our method was superior.

## Supporting Information

S1 File The Matlab implement for the MRMSn method. The compressed file includes the source code of MRMSn method and all the datasets in experiments.
(ZIP)
