# Supplementary material for: Gene Regulatory Network Inferences Using a Maximum-Relevance and Maximum-Significance Strategy
Source: PLoS One. 2016 Nov 9;11(11):e0166115. doi: 10.1371/journal.pone.0166115 (PMC5102470; doi:10.1371/journal.pone.0166115)
Supplement: S1 File — The compressed file includes the source code of MRMSn method and all the datasets in experiments. (ZIP) [file pone.0166115.s001.zip › The Matlab implement for the MRMSn method/Instruction for MRMSn.docx]

**Instruction for MRMSn**

**1. Introduction**

MRMSn is a novel regulatory network inference method based on MRMS strategy, which is performed in the MATLAB environment

**2. Matlab Code descriptions**

2.1 Folders

There are some subfolders in the folder “MRMSn ”.

“data” : the subfolder contains five expression dataset in our experiment.

“gold” : the subfolder contains five golden standard(benchmark) network.

“othermethodresult” : the subfolder contains the datasets which are the results of the other five inference method.

“result”: the subfolder contains the results of the dependence strength between given target gene and regulator gene ,which are used to calculate the AUC values.

2.2 mrms.m

[selectedgenes,dist] = mrms(k,Gval,n_gene,data,selnum,arf,bt)

This is the function code for select regulatory genes of the given target gene based on maximum-relevance and maximum-significance(MRMS)

2.2.1 Input:

| Input | Description |
| --- | --- |
| k | The index of the given target gene in the data |
| Gval | The mutual information matrix of variable |
| n_gene | The number of variable |
| data | The expression of variable, in which row is variable and column is the sample |
| selnum | The number of the selected regulator gene |
| arf | The parameter which is the weight of network relevance; |
| bt | The threshold of scoring |

2.2 .2 Output:

| Output | Description |
| --- | --- |
| selectedgenes | The regulatory genes of the given target gene |
| dist | The strength of dependence between given target gene and regulator gene |

2.3 arf_opt_value.m

arf= arf_opt_value(Gval,n_gene )

The function is used to choose the optimum values of , which is the weight of network relevance.

2.3.1 Input:

| Input | Description |
| --- | --- |
| Gval | The mutual information matrix of variable |
| n_gene | The number of variable |

2.3.2 Output:

| Output | Description |
| --- | --- |
| arf | The parameter which is the optimum weight of network relevance |

2.4 Test code based on optimum threshold

test_b3_small_chain.m: The file is used to infer gene regulatory network based on optimum threshold from Reaction chain with 4 species data.

test_10gene.m : The file is used to infer gene regulatory network based on optimum threshold from DREAM3 10 gene data.

test_50gene.m : The file is used to infer gene regulatory network based on optimum threshold from DREAM3 50 gene data.

test_b4_irma.m: The file is used to infer gene regulatory network based on optimum threshold from IRMA benchmark data.

test_sos.m : The file is used to infer gene regulatory network based on optimum threshold from S0S data.

2.5 Calculation of AUROC value of different methods on five datasets

resultAUCshow.m : show the AUC result of MRMSn for different dataset.

test_othermethod.m: Test code of other five methods ,which can show the running result and the AUC value on five datasets.

**3. Operation for Test code**

The files give the test codes for all the datasets. The operations of all the test codes have four steps:

1. Load files dataset including gene expression profiles and golden standard (benchmark) network.

2. Run the function “arf_opt_value.m” to obtain the value of arf, and set other parameters.

3. Run the function “mrms.m” and get the regulatory genes of each gene.

4. Refine the existing edges and get networks G_f.
