# Supplementary material for: Gene Regulatory Network Inferences Using a Maximum-Relevance and Maximum-Significance Strategy
Source: PLoS One. 2016 Nov 9;11(11):e0166115. doi: 10.1371/journal.pone.0166115 (PMC5102470; doi:10.1371/journal.pone.0166115)
Supplement: S1 File — The compressed file includes the source code of MRMSn method and all the datasets in experiments. (ZIP) [file pone.0166115.s001.zip › The Matlab implement for the MRMSn method/MRMSn/doc/Instruction for MRMSn.pdf]

Instruction for MRMSn

1. Introduction

MRMSn is a novel regulatory network inference method based on MRMS strategy, which is performed in the MATLAB environment

2. Matlab Code descriptions

2.1 Folders

There are some subfolders in the folder “MRMSn ”.

“data” : the subfolder contains five expression dataset in our experiment.

“gold” : the subfolder contains five golden standard(benchmark) network.

“othermethodresult” : the subfolder contains the datasets which are the results of the other five inference method.

“result”: the subfolder contains the results of the dependence strength between given target gene and regulator gene ,which are used to calculate the AUC values.

2.2 mrms.m

```
[selectedgenes,dist] = mrms(k,Gval,n_gene,data,selnum,arf,bt)
```

This is the function code for select regulatory genes of the given target gene based on maximum-relevance and maximum-significance(MRMS)

2.2.1 Input:

Input	Description
**k**	The index of the given target gene in the data
**Gval**	The mutual information matrix of variable
**n_gene**	The number of variable
**data**	The expression of variable, in which row is variable and column is the sample
**selnum**	The number of the selected regulator gene
**arf**	The parameter which is the weight of network relevance;
**bt**	The threshold of scoring

2.2 .2 Output:

Output	Description
**selectedgenes**	The regulatory genes of the given target gene
**dist**	The strength of dependence between given target gene and regulator gene

2.3 arf_opt_value.m

```
arf= arf_opt_value(Gval,n_gene )
```

The function is used to choose the optimum values of α, which is the weight of network relevance.

2.3.1 Input:

Input	Description
**Gval**	The mutual information matrix of variable
**n_gene**	The number of variable

2.3.2 Output:

Output	Description
**arf**	The parameter which is the optimum weight of network relevance

2.4 Test code based on optimum threshold

test_b3_small_chain.m: The file is used to infer gene regulatory network based on optimum threshold from Reaction chain with 4 species data.

test_10gene.m : The file is used to infer gene regulatory network based on optimum threshold from DREAM3 10 gene data.

test_50gene.m : The file is used to infer gene regulatory network based on optimum threshold from DREAM3 50 gene data.

test_b4_irma.m: The file is used to infer gene regulatory network based on optimum threshold from IRMA benchmark data.

test_sos.m : The file is used to infer gene regulatory network based on optimum threshold from S0S data.

2.5 Calculation of AUROC value of different methods on five datasets

resultAUCshow.m : show the AUC result of MRMSn for different dataset.

test_othermethod.m: Test code of other five methods ,which can show the running result and the AUC value on five datasets.

3. Operation for Test code

The files give the test codes for all the datasets. The operations of all the test codes have four steps:

1. Load files dataset including gene expression profiles and golden standard (benchmark) network.
2. Run the function “arf_opt_value.m” to obtain the value of arf, and set other parameters.
3. Run the function “mrms.m” and get the regulatory genes of each gene.
4. Refine the existing edges and get networks G_f.
